# Supplementary material for: Failure without Tears: Two-Step Attachment in a Climbing Cactus
Source: Biomimetics (Basel). 2023 May 25;8(2):220. doi: 10.3390/biomimetics8020220 (PMC10296533; doi:10.3390/biomimetics8020220)
Supplement: Supplementary file 1 [file biomimetics-08-00220-s001.zip › biomimetics-2299429-supplementary/Supplementary Materials Descriptions.pdf]

Supplementary Materials Descriptions

Video S1. Spine attachment in a wind-prone habitat. The Selenicereus setaceous grows in several coastal habitats, including wind-prone, shrubby vegetation along the coast of eastern Brazil. This habitat experiences almost constant wind-blown conditions. In these conditions, the cactus depends on the deployment of recurved spines (step 1 attachment) to anchor the climbing stems among dense branches of shrubs.
